# Efficient Method for Molecular Characterization of the 5′ and 3′ Ends of the Dengue Virus Genome

**DOI:** 10.3390/v12050496

**Published:** 2020-04-29

**Authors:** Alicia Rosales-Munar, Diego Alejandro Alvarez-Diaz, Katherine Laiton-Donato, Dioselina Peláez-Carvajal, Jose A. Usme-Ciro

**Affiliations:** 1 Sequencing and Genomics Unit, Virology Laboratory, Dirección de Redes en Salud Pública, Instituto Nacional de Salud, Bogotá 111321, Colombia; aliciaarosalesm@gmail.com (A.R.-M.); dalvarezd@ins.gov.co (D.A.A.-D.); kdlaiton@ins.gov.co (K.L.-D.); dpelaez@ins.gov.co (D.P.-C.); 2 Centro de Investigación en Salud para el Trópico-CIST, Facultad de Medicina, Universidad Cooperativa de Colombia, Santa Marta 470003, Colombia

**Keywords:** genome ends, dengue virus, RACE-PCR, poly(A) polymerase

## Abstract

Dengue is a mosquito-borne disease that is of major importance in public health. Although it has been extensively studied at the molecular level, sequencing of the 5′ and 3′ ends of the untranslated regions (UTR) commonly requires specific approaches for completion and corroboration. The present study aimed to characterize the 5′ and 3′ ends of dengue virus types 1 to 4. The 5′ and 3′ ends of twenty-nine dengue virus isolates from acute infections were amplified through a modified protocol of the rapid amplification cDNA ends approach. For the 5′ end cDNA synthesis, specific anti-sense primers for each serotype were used, followed by polyadenylation of the cDNA using a terminal transferase and subsequent PCR amplification with oligo(dT) and internal specific reverse primer. At the 3′ end of the positive-sense viral RNA, an adenine tail was directly synthetized using an *Escherichia coli* poly(A) polymerase, allowing subsequent hybridization of the oligo(dT) during cDNA synthesis. The incorporation of the poly(A) tail at the 5′ and 3′ ends of the dengue virus cDNA and RNA, respectively, allowed for successful primer hybridization, PCR amplification and direct sequencing. This approach can be used for completing dengue virus genomes obtained through direct and next-generation sequencing methods.

## 1. Introduction

*Dengue virus* (DENV), the etiological agent of dengue fever, has been considered one of the most important vector-borne viruses in the tropical world, where an estimated of 390 million infections occur each year [1, 2,3]. A high proportion (around 75%) of DENV infections are asymptomatic, with the remaining 50–100 million infections ranging from dengue without/with warning signs to severe dengue, a life-threatening condition requiring precise clinical management [2].

DENV is a serocomplex of four closely related serotypes (DENV-1 to -4), belonging to the family *Flaviviridae,* genus *Flavivirus* [4,5]. The genome consists of a positive-sense single-stranded RNA of approximately 11 kb, which encodes for three structural (capsid, pre-membrane and envelope) and seven nonstructural (NS1, NS2A, NS2B, NS3, NS4A, NS4B, and NS5) proteins. At the 5′ and 3′ untranslated regions (UTR), DENV contains several evolutionarily conserved structural RNA elements involved in viral protein synthesis, genome replication, the regulation of viral and host processes and pathogenesis [6].

The 5′ UTR has a length of 95 to 101 nucleotides and contains two structural domains, the stem-loop A (SLA) domain directly interacts with the viral RNA-dependent RNA polymerase (RdRp) [7], while the stem-loop B (SLB) contains a 16-nucleotide element (5′ Upstream AUG Region-5′ UAR), that together with other RNA elements and structures located in the capsid-coding region (5′ Downstream AUG Region-5′ DAR and 5′ cyclization sequence-5′ CS) are involved in genome cyclization promoting viral RNA synthesis [8,9]. Other RNA elements downstream the AUG region are critical for viral functions; the capsid hairpin (cHP) participates in viral protein translation and RNA synthesis and the conserved capsid-coding region 1 (CCR1) is responsible for virus assembly and viral particle production [10,11].

The 3′ UTR, lacking a poly(A) tail, contains evolutionarily conserved RNA structures and regulatory elements involved in the virus-host relationship. It has been divided into three regions (I–III). Region I contains two stem-loops structures (SLI and SLII) located downstream the stop codon of the viral polyprotein; region II contains two dumbbell structures (DB1 and DB2); and region III contains two RNA structures, sHP and the highly conserved 3′ stem-loop structure (3′ SL), with approximately 79 nucleotides in length at the end of the genome [12]. Several cis-acting elements in the 3′ UTR, including pseudoknots (PKI, PKII, PKIII and PKIV) and sequences complimentary to the 5′ region leading to viral RNA circularization (3′CS, 3′DAR, 3′UAR) have been described [9]. They have important roles in modulating viral replication and host processes [13].

DENV genome has been extensively characterized at the molecular level, which is crucial for comparative analyses attempting to identify genetic determinants of disease severity and dengue pathogenesis. However, complete and corroborated sequencing of the 3′ end of single-stranded positive sense viral RNA genomes lacking a poly(A) tail represents a challenge. Technical approaches of most of the commercially available kits for characterization of the 3′ ends are indicated for poly(A)-tailed messenger RNA through 3′ random amplification of cDNA ends-PCR (RACE-PCR). Some modifications using the enzymatic activity of T4 RNA ligase and poly(A) polymerase have been previously described for viral and bacterial models, respectively [14,15]. Here, we describe the successful implementation of terminal transferase-based 5′ RACE-PCR and poly(A) polymerase-based 3′ RACE-PCR approaches for extension and sequencing of the ends of the DENV genome.

## 2. Materials and Methods

### 2.1. Cell Cultures, Viral Isolation and Titration

Human sera from DENV-infected patients were diluted (1/100) in minimal essential medium (MEM) supplemented with 1% HEPES, 1% NaHCO_3_, 5% Tryptose and 2% fetal bovine serum. Subsequently, C6/36 cell cultures were inoculated with 200-µL of each diluted serum. The adhesion step was carried out at 28 °C for 1 h, then 800 µL of MEM supplemented with 2% fetal bovine serum was added. The cultures were incubated at 28 °C and the supernatants collected when cytopathic effect (CPE) was observed (typically syncytia or cell growth arrest), or 9 days post-infection. A second passage for samples without showing CPE was performed by transferring a 200-µL aliquot of the cell supernatant from the first passage to a fresh C6/36 culture. Culture supernatants were collected, cleared, aliquoted and stored at −70 °C. Virus titer was obtained by plaque assay in BHK-21 (ATCC, CRL-12071 ™) monolayers. For this, 7 serial dilutions 1:10 of the culture supernatant were made in MEM. Next, 200 µL of each dilution was added to BHK-21 cells grown in 24-well dishes, at a density of 2 × 10^5^ cells/well and incubated at 37 °C, 5% CO_2_ for 1 h. Subsequently, 1.5 mL of 1.5% carboxymethyl-cellulose (CMC) in MEM supplemented with 2% FBS was added and incubated for 8 days at 37 °C in 5% CO_2_, to allow plaque formation. For plaque counting, the cell monolayers were stained with 0.2% crystal violet solution in 3.5% formaldehyde and 35% ethanol. DENV titer was expressed as plaque-forming units (PFU)/mL.

### 2.2. Viral RNA Extraction and DENV Serotyping

Viral RNA was extracted from 140 μL of culture supernatants using the QIAamp Viral RNA Mini Kit (Qiagen Inc., Chatsworth, CA, USA), following the manufacturer’s recommendations. Purified RNA was stored at −70 °C for subsequent applications. DENV serotyping was performed through a conventional fourplex RT-PCR, as previously described [16].

### 2.3. Primer Design for Amplification of the DENV 5′ and 3′ Ends of the DENV Genome

The 5′/3′ RACE kit 2nd generation (Roche Diagnostics GmbH, Mannheim, Germany) was used by following the manufacturer’s recommendations. Briefly, three specific antisense primers (SP1, SP2 and SP3) and two sense primers (SP4 and SP5) were designed for cDNA synthesis and PCR amplification of the 5′ and 3′ ends of all DENV serotypes. Primer design was carried out through the PrimerSelect module of the LaserGene^®^ suite version 8.1 (DNASTAR Inc., Madison, WI, USA.). Genomic sequences GQ868568 (DENV-1), NC_001474 (DENV-2), GU131954 (DENV-3) and GQ868585 (DENV-4) were used as reference. Once the best primer sets were defined (Table 1), homologous sequences of the different serotypes and genotypes of DENV circulating in the Americas were aligned through the ClustalW algorithm implemented in MEGA version 6 [17], in order to assess and consider the genetic variability from each genomic region. Therefore, the design was limited to the circulating genotypes in the Americas, which have been stable through time and geographic distribution [18]. The remaining genotypes were not included, due to the high divergence and limitations for primers degeneracy. Primers were designed by following these criteria: (1) length between 17–24 mer; (2) melting temperature between 48–60 °C; (3) stability of the 3′ pentamer −8,5 Kcal/mol; (4) 7-bp length unique sequence in the 3′ of the primer, (5) free 3′-OH of the primer should be complementary to a second or first codon position (if located in a coding region); (6) inclusion of degenerate sites for those nucleotide positions with representative genetic variability among strains through the region. Degenerated sites were introduced according to the IUPAC nucleotide ambiguity code, available at: [19].

### 2.4. Reverse Transcription and PCR Amplification of the 5′ Region of the DENV Genome

**Figure 1:**
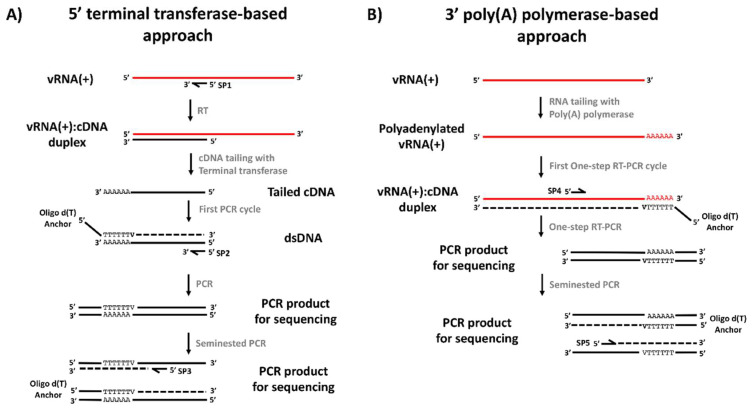
General strategy for viral genome ends’ amplification and sequencing. (**A**) Terminal transferase-based protocol for amplification of the 5′ end of the viral genome. (**B**). Poly(A) polymerase-based protocol for amplification of the 3′ end of the viral genome. V: A, C or G.

The DENV 5′ end cDNA covering the 5′UTR and part of the coding region was synthesized using the GoScript Reverse Transcription System (GoScript-Promega, Madison, USA), following the manufacturer’s recommendations (Figure 1A). The reaction mixture included between 100–200 ng of total RNA extract, serotype specific anti-sense primer SP1 (0.625 μM), 500 µM dNTPs, 25 U reverse transcriptase, MgCl_2_ (2.6 mM),1X cDNA synthesis buffer, 5 U RNaseOUT (Life Technologies, Carlsbad, CA, USA) and nuclease-free water, for a final volume of 20 μL. The mixture was incubated for 60 min at 55 °C, then, reverse transcriptase was inactivated by heating at 85 °C for 5 min. The purification of cDNA products was performed using the QIAquick PCR purification kit (Qiagen Inc., Chatsworth, CA, USA) following the manufacturer’s recommendations. Poly (A) tailing of the first-strand cDNA using a recombinant terminal transferase and PCR amplification of dA-tailed cDNA were performed according to the 5′/3′ RACE kit 2nd generation manufacturer’s instructions. The PCR was carried out with the serotype-specific primer SP2 and the oligo d(T) anchor primer (5′-GACCACGCGTATCGATGTCGACTTTTTTTTTTTTTTTTV3′). The reaction mix contained 5 μL of the polyadenylated cDNA, SP2 (0.25 μM), oligo d(T) anchor primer (0.75 μM), 1.25 U of *Taq* DNA polymerase (Life Technologies Corp., Carlsbad, CA, USA), dNTPs (0.4 mM), MgCl_2_ (2 mM), 5 μL of 1× buffer without Mg^2+^ and nuclease-free water, for a final volume of 50 μL. The thermal profile consisted of a denaturation step at 94 °C for 5 min, followed by 40 cycles of (94 °C for 15 s, primer-specific annealing temperature for 30 s, 72 °C for 30 s), followed by a final extension at 72 °C and 5 m. Expected sizes were 453, 380, 448 and 477 bp for DENV-1 to -4, respectively. Subsequently, a nested PCR was performed using the PCR Anchor primer (5′-GACCACGCGTATCGATGTCGAC-3′) and serotype-specific SP3 reverse primer located upstream of SP2. The reaction mixture included 0.5 μL of the SP2-Oligo d(T) PCR product, SP3 (0.25 μM), anchor primer (0.25 μM), *Taq* DNA polymerase (1.25 U), dNTPs (400 µM), MgCl_2_ (2 mM) and 5 mL of 10× buffer, for a final volume of 50 μL. The thermal profile was the same described above. Expected sizes were 310, 300, 298, and 321 bp for DENV-1 to -4, respectively.

### 2.5. Poly (A) Tailing, Reverse Transcription and PCR Amplification of the End of 3′ Untranslated Region

Given that RNA extracts from infected cell supernatants contain almost exclusively positive-sense DENV genomes and the DENV genome lacks a poly (A) tail, it was not possible to use specific forward primers (SP4 or SP5) or oligo-dT for cDNA synthesis. Therefore, two strategies were defined. The first one consisted in using total RNA obtained from lysates of DENV-infected C6/36 cells (in order to obtain minus strands of the viral RNA) to perform the classic RACE-PCR. The second one was based on the direct A-tailing of positive-sense DENV RNA genomes with 3′-adenine residues, allowing the subsequent annealing of the oligo-dT Anchor Primer (Figure 1B). Briefly, 15 µL of total RNA (~500 ng) were poly-adenylated at the 3′ end in a reaction mixture containing 0.5 U of *E. coli* poly(A) polymerase system (New England Biolabs, Ipswich, MA, USA), *E. coli* poly(A) polymerase reaction buffer (50 mM Tris-HCl, 250 mM NaCl, 10 mM MgCl_2_) and 1mM adenosine-5′-triphosphate (ATP), followed by incubation at 37 °C for 30 min. Afterwards, 5 µL from the reaction were used for a one step RT-PCR using the SuperScript™ III One-Step RT-PCR System (Life Technologies, Carlsbad, CA, USA). Briefly, 50 μL of the reaction were prepared by mixing 25 μL of 2× Reaction Mix, 1 μL of oligo dT-Anchor primer (37.5 μM), 1 μL of specific primer SP4 (12.5 μM), 1 μL of SuperScript™ III RT/Platinum™ *Taq* Mix, 1 μL of RNaseOUT (5 U/μL) 5 µL of A-tailed RNA and H_2_O to 50 µL. The thermal profile followed the manufacturer’s recommendations and primer specific annealing temperature. Finally, if no amplification was observed, a nested PCR was performed using the serotype-specific SP5 and the oligo-dT-Anchor primer, with the same conditions described above for the 5′ untranslated region. The expected sizes of the PCR products generated with the SP4 primer were 713, 563, 490 and 452 bp for DENV-1 to -4, respectively; while those for the SP5 primer were 581, 438, 389 and 389 bp for DENV-1 to -4, respectively.

### 2.6. DNA Sequencing and Sequence Handling

The purification of PCR amplicons was performed using the QIAquick PCR purification kit (Qiagen Inc., Chatsworth, CA, USA), following the manufacturer’s recommendations. For sequencing of the genome ends, reaction mixtures were prepared with 10 ng of each purified PCR product, 2 μL of BigDye Terminator Cycle Sequencing v 3.1 (Applied Biosystems, Carlsbad, CA, USA), 3.2 pmol/μL of the serotype-specific oligonucleotide and 2.0 μL of 5× Sequencing buffer in a final volume of 10 μL. The thermal profile consisted of an initial denaturation at 96 °C for 1 min, followed by 25 cycles of denaturation at 96 °C for 10 s, hybridization at 50 °C for 5 s and extension at 60 °C for 4 min. The products of the sequencing reactions were purified with the BigDye XTerminator purification kit (Applied Biosystems, Carlsbad, CA, USA) and sequenced on the ABI3130 Genetic Analyzer (Applied Biosystems, Carlsbad, CA, USA). The sequences were edited and assembled into the SeqMan module of the LaserGene v8.1 suite (DNASTAR Inc. Madison, WI, USA).

## 3. Results

### 3.1. Successful Amplification of the 5′ and 3′ Regions of All DENV Serotypes

The 5′ end of Colombian strains corresponding to the four DENV serotypes was successfully amplified by using the standard RACE-PCR approach, that involved the polyadenylation of the cDNA generated with the primer SP1 and subsequent PCR amplification. DENV-2 and -3 were amplified after the first round of PCR amplification with serotype-specific primers SP2, while bands of the expected size were observed for all DENV serotypes after the second round of PCR amplification with serotype-specific primers SP3 (Supplementary Figure S1).

The amplification of the DENV 3′ end was attempted by a similar RACE-PCR strategy, using total RNA extracts from DENV-infected C6/36 cells and forward serotype-specific SP4 primers, which were expected to hybridize the minus strand viral RNA for the cDNA synthesis. Subsequent PCR was unsuccessful, probably due to the asymmetric replication of the DENV genome, and therefore the low abundance of negative-sense viral RNA strands [20]. A second approach based on the poly-adenylation of positive-sense viral RNA by using the *E. coli* poly (A) polymerase, and subsequent oligo-dT hybridization on the artificially generated poly (A) tail, demonstrated to be successful for the specific amplification of the 3′ ends of all DENV serotypes (Supplementary Figure S2).

### 3.2. Molecular Characterization of the 5′ and 3′ Ends of the Dengue Virus Genome

**Figure 2:**
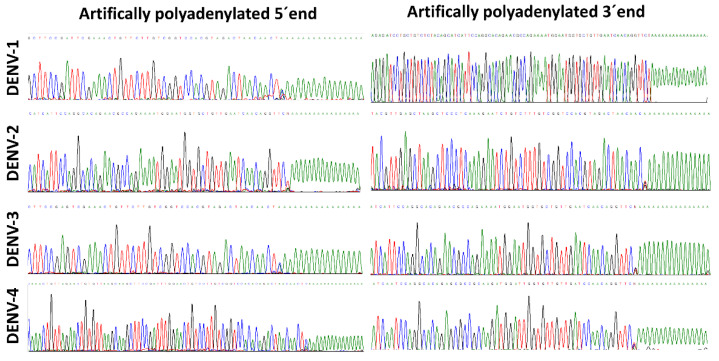
Artificial polyadenylation at the 5′ and 3′ ends of the dengue virus genome. Electropherograms of the 5′and 3′ ends of representative strains of DENV-1 to DENV-4, obtained through Sanger sequencing. The 5′ and 3′ ends were sequenced by using sequence-specific reverse and forward primers, respectively.

The DENV 5′ and 3′ ends of all four DENV serotypes were successfully obtained after Sanger sequencing with specific primers (SP2, SP3, SP4 and SP5). A total of 29 isolates from Colombian clinical samples were successfully sequenced, 26 isolates belonging to patients with dengue without warning signs (3, 10, 10 and 3 from DENV-1 to -4, respectively) and 5 from severe dengue patients (all from DENV-2) (Table 2). Viral titers ranged from 1 × 10^4^ to 2 × 10^7^ PFU/mL. The electropherograms allowed one to confirm the presence of the artificially generated poly (A) tail (Figure 2). At the 3′ end, the last nucleotide is called “N” to designate a variable position; this is a consequence of using a degenerate oligo-dT anchor primer containing A, C or G at the 3′ end. At the 5′ end, the “N” is also present at the -1 position, therefore, it does not affect the sequence of interest.

### 3.3. Complete Coverage at the 5′ and 3′ Ends of the DENV Genome

**Figure 3:**
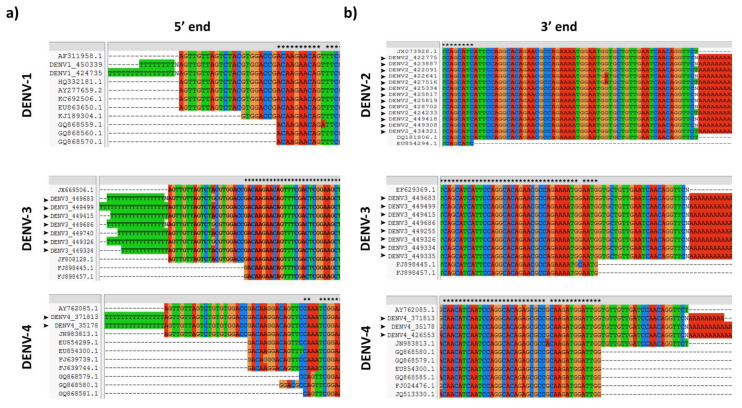
DENV 5′ and 3′ ends sequence coverage. (**A**) Alignment of DENV 5′ end sequences obtained in the present study, with some records of complete genomes available at GenBank. (**B**) Alignment of DENV 3′ end sequences obtained in the present study, with some records of complete genomes available at GenBank. Black arrowheads denote sequences obtained in the present study.

In order to assess the coverage at the 5′ and 3′ ends, previously reported sequences of DENV strains circulating in South American countries were aligned with those of the present study. A high proportion of DENV “complete genome” records available at GenBank have incomplete or uncorroborated 5′ and 3′ ends, commonly lacking the first or last ~20 nt at the 5′ and 3′ UTR, respectively, due to limitations imposed by current RNA genome amplification techniques by reverse transcription and PCR, which involves the use of primers with previously known sequences during cDNA synthesis, PCR amplification or sequencing. These ends were completely covered by using the strategy described here (Figure 3). Using the BLAST alignment tool (http://blast.ncbi.nlm.nih.gov/Blast.cgi), the sequences obtained in the present study matched with other DENV sequences reported in Latin American and Caribbean countries (Cuba, Haiti, Brazil, Peru, Venezuela, Ecuador, French Guiana, Argentina, Chile and Paraguay).

## 4. Discussion

During the last two decades, the intense research in fundamental biology of DENV and other closely related flaviviruses has allowed one to decipher the role or function of almost all viral proteins and RNA structural elements during the virus life cycle [11]. These studies have also allowed the identification of several genetic differences that explain some of the phenotypic differences at the virus replication or virus-host interaction and pathogenesis levels, including nucleotide substitutions and insertion/deletions in the coding and the untranslated regions [21,22]. The 5′ and 3′ UTR harbor critical elements for viral RNA replication and translation becoming putative candidates to virulence determinants [23,24,25,26]; for this reason, complete sequencing, including the genome ends, is mandatory for identifying the viral genetic contribution to virulence and severe dengue pathogenesis.

Viral RNA polymerases are error-prone and lack proofreading activity, leading RNA viruses to exhibit extremely high mutation rates [27]. The unavoidable accumulation of genetic variation in the whole DENV genome suppose a challenge for sequence-based molecular methods and therefore justify the rational design of degenerated oligonucleotides that theoretically cover the existent genetic variability and perform well for a longer period of mutation accumulation. Here, we used the PrimerSelect module of the LaserGene suite, which integrates the nearest neighbor algorithm for ΔG and melting temperature (Tm, temperature at which half of the oligonucleotides have hybridized with the sequence) calculation. Using this algorithm, it was possible to design primers that worked well for amplification and sequencing the 5′ and 3′ ends of the DENV genome. All primers were analyzed through sequence alignment to cover the existing variability of DENV strains across the region and to avoid the use of critical primer positions according to evolutionary behavior (e.g., codon position in coding regions).

Some authors have described the complete DENV genome sequencing, despite imposing the primer sequence at the end of the 5′ and 3′ ends [28,29,30]. Other authors have recognized the limitation of the genome ends characterization and have limited their published genomes to actually characterized nucleotides, excluding the primer sequences [31].

The most rigorous approach is well-known as RACE-PCR. The original RACE protocol for the 3′ end was intended to work directly with polyadenylated RNA (e.g., messenger RNA), however, flavivirus genomes lack a poly(A) tail. A first approach to overcome this limitation consisted of applying the RACE strategy to total RNA extracts obtained from cell lysates, in which the presence of negative-sense viral RNA within dsRNA and replicative intermediates was expected. However, the asymmetric replication of the DENV genome and the low abundance of negative-sense viral RNA strands [20] impeded the success of this strategy. Therefore, an approach for the 3′ end characterization was implemented by using the *E. coli* poly (A) polymerase, which was based on the direct polyadenylation of the positive-sense viral RNA genomes previously extracted from viral particles present in culture supernatants of infected cells. Despite the fact that cellular mRNA transcripts could be present in the extract after viral RNA extraction, the use of virus-specific forward primer after the cDNA synthesis for PCR amplification prevents any interference. The artificially generated poly (A) tail provided a target for the oligo-dT hybridization during the cDNA synthesis and subsequent PCR amplification, following the standard RACE protocol. High quality electropherograms evidenced the successful incorporation of a poly (A) tract to the viral genome and the complete resolution of the 3′ end sequence of the analyzed DENV strains from all four serotypes and from a wide range of virus concentrations.

Novel protocols for the Oxford Nanopore sequencing of RNA virus genomes in their native form have been recently described [32,33]. They employed different approaches for obtaining the starting material and share the artificial polyadenylation step to extend the 3′ end of the viral genome before sequencing. These methods have not been validated in extreme cases of very low RNA concentration or RNA fragmentation/degradation, where a previous amplification step has been shown to improve the performance. Next generation sequencing (NGS) protocols for non-polyadenylated RNA viruses involving amplification require previous knowledge of the sequence for primer hybridization. Therefore, we are extending the method as an easy and useful strategy for genome ends characterization, where PCR amplification is required.

These technical approaches could be implemented in basic molecular biology laboratories and routinely used as a complement for full-genome sequencing of DENV and the general approach may be useful for molecular characterization of the genome ends of whatever virus species with a linear RNA genome. This strategy is compatible with Sanger sequencing or NGS, increasing the coverage at the genome ends and allowing more accurate in comparative genomics studies.

## 5. Conclusions

The methodological approach was successful for the amplification and sequencing of the 5′ and 3′ ends of the DENV-1 to -4 genomes. The application of this strategy will allow the evaluation of the effect of point mutations on the disruption/conservation of critical RNA structural elements and their potential impact on viral replication and pathogenesis.

## Figures and Tables

**Table 1:** Serotype-specific primers used for amplification and sequencing of the 5′ and 3′ ends of the *Dengue virus* genome.

Serotype	Primer Name	Region	Genomic Position ^a^	Sequence (5′-3′) ^b^	Annealing T °C
DENV-1	DENV1_SP1	5′	560–579	ccgggggcatttgtaggtca	50 for cDNA
	DENV1_SP2	5′	431–453	tatcatgtgtggctctccycctc	50
	DENV1_SP3	5′	287–310	ctttgatygctccattcttcttga	58
	DENV1_SP4	3′	9949–9970	caccaatggatgacaacagaag	55
	DENV1_SP5	3′	10,119–10,140	cacctgggccaccaacatacaa	55
DENV-2	DENV2_SP1	5′	475–497	cctttctcctgcctaccaacgat	50 for cDNA
	DENV2_SP2	5′	355–376	tgttcagcatccttccaatctc	50
	DENV2_SP3	5′	287–310	tcgttccccatcttttyagtatcc	50
	DENV2_SP4	3′	10,114–10,137	agaacatccaaacagcaataaatc	52
	DENV2_SP5	3′	10,238–10,262	aagggaagaggaagaggcaggtgt	52
DENV3	DENV3_SP1	5′	560–579	ggtratrtgggggcatttgtaag	50 for cDNA
	DENV3_SP2	5′	429–448	cggctctccatctcgtgaag	50
	DENV3_SP3	5′	276–298	cgacttcttgaaggttccccatc	58
	DENV3_SP4	3′	10,210–10,233	gaaggaggaggartcggaggg	55
	DENV3_SP5	3′	10,311–10,333	gcctgtgagccccgtctaag	50
DENV-4	DENV4_SP1	5′	496–517	ttgttgatcccctctgttgtyt	50 for cDNA
	DENV4_SP2	5′	454–477	ccctttcatgttttgccactatca	50
	DENV4_SP3	5′	298–321	gtgggatggaaagractcgca	55
	DENV4_SP4	3′	10,302–10,320	gcaaaccgtgctgcctgta	52
	DENV4_SP5	3′	10,098–10,120	ggactttcttcyagagccacctg	52

^a^ Genomic positions were estimated for every DENV serotype according to the sequences available in GenBank with accession numbers: GQ868568 (DENV-1), NC_001474 (DENV-2), GU131954 (DENV-3) and GQ868585 (DENV-4). ^b^ Degenerate sites were included by following the IUPAC nucleotide ambiguity code.

**Table 2:** List of DENV-1, -2, -3 and -4 strains included in the study and associated clinical characteristics.

Serotype	Internal Code	Year	State	Clinical Classification	Virus titer (PFU/mL)
DENV 1	424735	2013	Meta	D	7.8 × 10^6^
	427493	2013	Tolima	D	6.83 × 10^6^
	450339	2015	Cundinamarca	D	4.33 × 10^5^
	483718	2016	Cauca	D	9.75 × 10^5^
	484940	2016	Boyaca	D	6.83 × 10^4^
DENV 2	423887	2013	Putumayo	D	1.2 × 10^5^
	427516	2013	Caldas	SD	1.7 × 10^7^
	425334	2013	Putumayo	D	2 × 10^7^
	425817	2013	Tolima	D	2 × 10^7^
	425819	2013	Cundinamarca	D	1.8 × 10^7^
	428702	2014	Tolima	SD	8.17 × 10^6^
	424233	2013	Boyacá	D	8.5 × 10^6^
	449418	2015	Tolima	DWS	8.83 × 10^6^
	449308	2015	Huila	SD *	2.83 × 10^4^
	449618	2015	Huila	D	1.5 × 10^6^
	449510	2015	Putumayo	D	1.48 × 10^6^
	434321	2014	Meta	SD	1.6 × 10^7^
DENV 3	449683	2015	Risaralda	DWS	1.9 × 10^7^
	449499	2015	Risaralda	D	1.72 × 10^6^
	449415	2015	Putumayo	D	NA
	449740	2015	Sucre	D	NA
	449746	2015	Risaralda	D	1 × 10^4^
	449255	2015	Quindío	D	1.23 × 10^5^
	449326	2015	Risaralda	D	7.33 × 10^6^
	449334	2015	Boyacá	DWS	NA
	449335	2015	Boyacá	DWS	8.75 × 10^6^
DENV 4	371813	NA	NA	NA	NA
	37178	NA	NA	NA	NA
	426553	2013	Risaralda	D	1.03 × 10^4^

NA: Not available. D: Dengue without warning signs. DWS: Dengue with warning signs. SD: Severe dengue. * Fatal case.

## Supplementary Materials

The following are available online at https://www.mdpi.com/1999-4915/12/5/496/s1, Figure S1: PCR amplification of the 5′end of DENV-1 to -4, Figure S2: PCR amplification of the 3′end of DENV-1 to -4.
