# Supplementary material for: Efficient Method for Molecular Characterization of the 5′ and 3′ Ends of the Dengue Virus Genome
Source: Viruses. 2020 Apr 29;12(5):496. doi: 10.3390/v12050496 (PMC7290889; doi:10.3390/v12050496)
Supplement: Supplementary file 1 [file viruses-12-00496-s001.pdf]

Supplementary figures

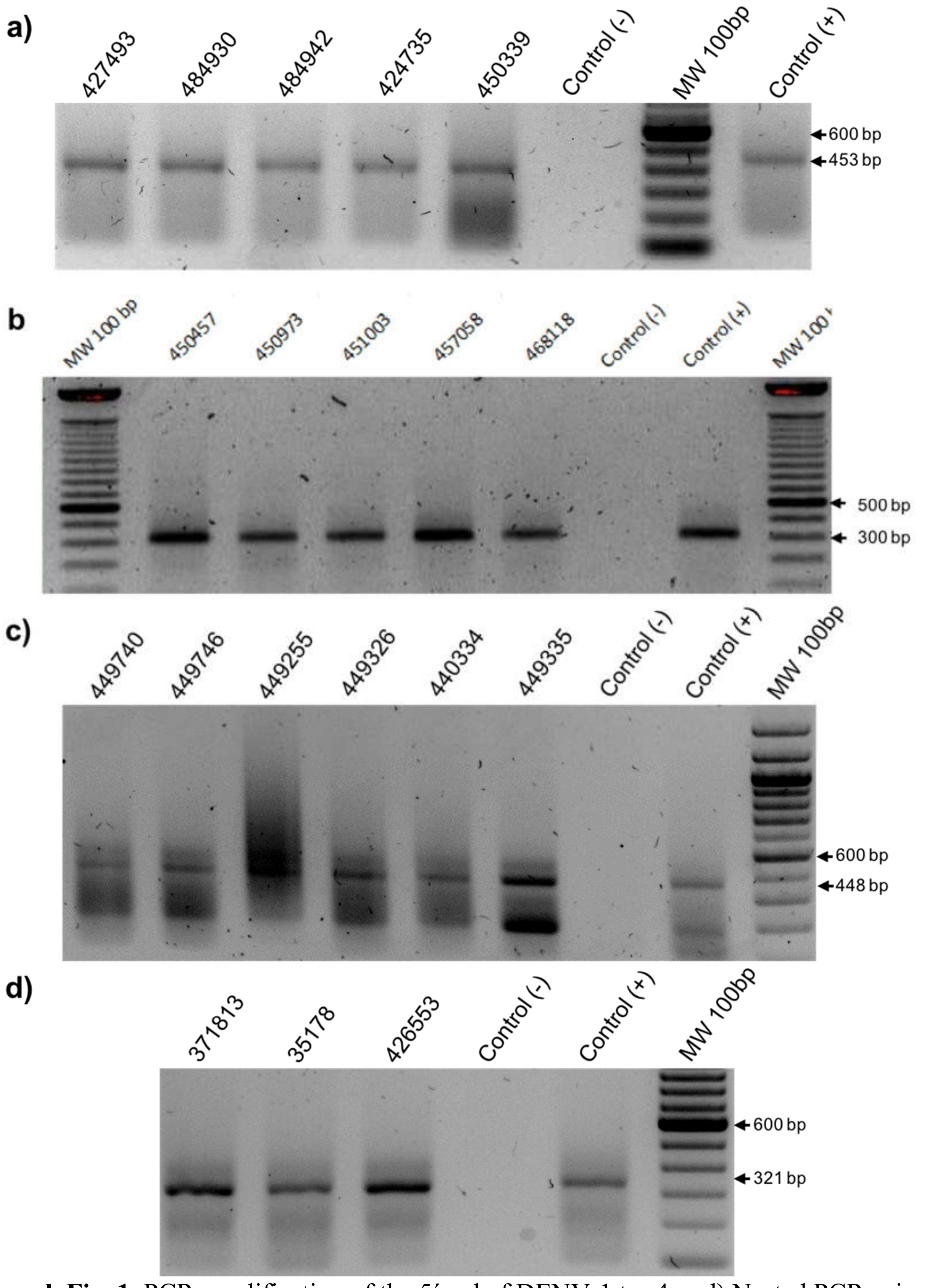

Suppl. Fig. 1. PCR amplification of the 5´end of DENV-1 to -4. a-d) Nested PCR using SP2 or SP3 and oligodT anchor primers for successful amplification of DENV strains. Previously characterized DENV strains were used as positive controls. The PCR product was 453 (SP2), 300 (SP3) 448 (SP2) and 321 (SP3) bp in length for DENV-1 to -4, respectively. MW: 100 bp DNA ladder (Invitrogen cat # 15628019 and Thermo Fisher Scientific cat # SM0323).

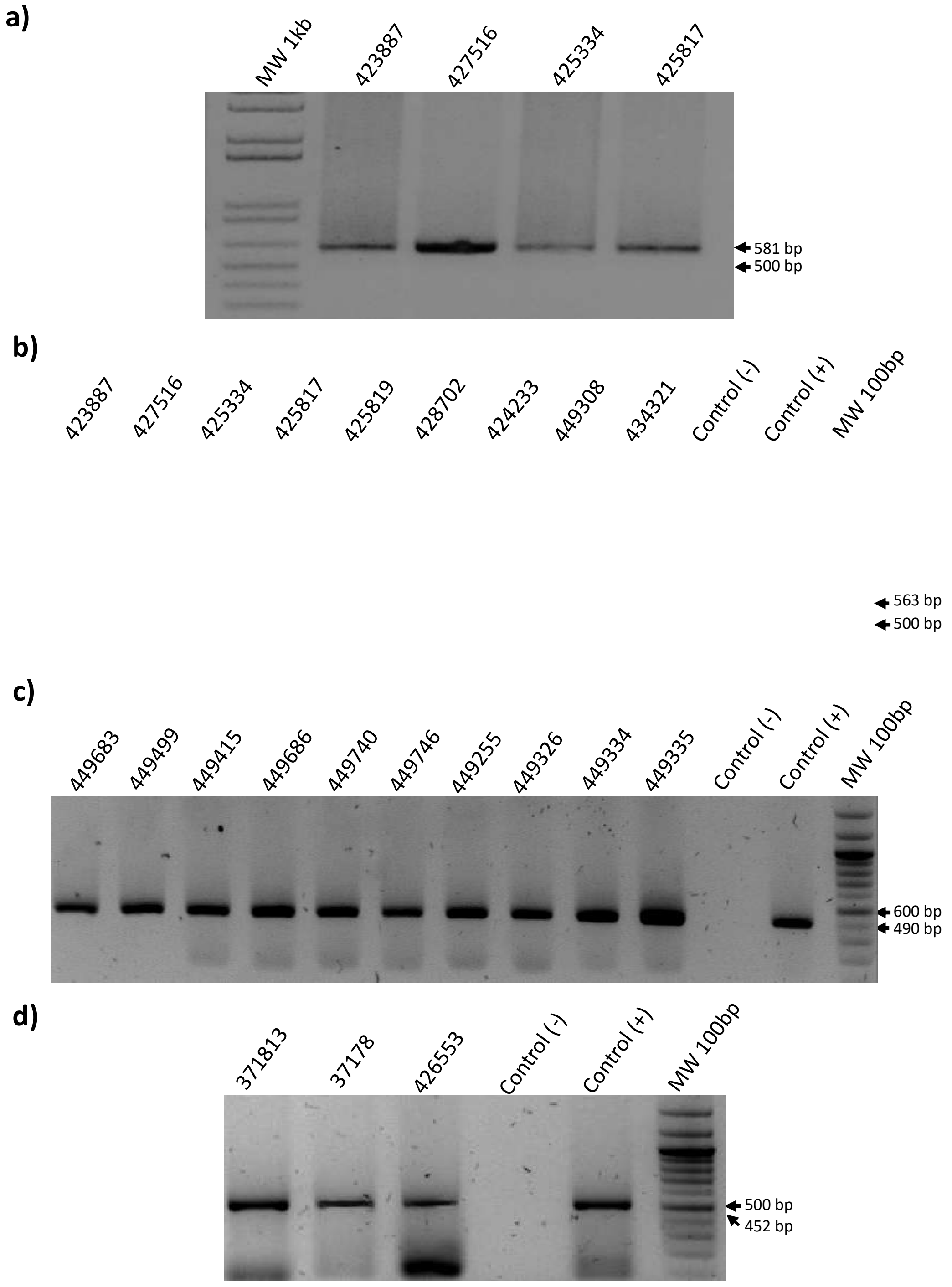

Suppl. Fig. 2. PCR amplification of the 3´end of DENV-1 to -4. a), b) and c). Nested PCR using SP4 and oligodT anchor primers for successful amplification of DENV-2, -3 and -4 strains, respectively. Previously characterized DENV strains were used as positive controls. The PCR product was 563, 490 and 452 bp in length for DENV-2, -3 and -4, respectively. MW: MW: 100 bp and 1 kb DNA ladder (Invitrogen cat # 15628019 and 10787026).
